# Practice Patterns of Graduates of a Rural Emergency Medicine Training Program

**DOI:** 10.5811/westjem.18661

**Published:** 2024-11-20

**Authors:** Dylan S. Kellogg, Miriam S. Teixeira, Michael Witt

**Affiliations:** * Arnot Ogden Medical Center, Department of Emergency Medicine & Emergency Medicine Residency, Elmira, New York; † Arnot Ogden Medical Center, EMS Education Program, Elmira, New York; ‡ Arnot Ogden Medical Center, Research Division, Department of Graduate Medical Education, Elmira, New York

## Abstract

**Introduction:**

Rural communities continue to face a shortage of emergency physicians despite the growing number of emergency medicine (EM) residencies. In rural areas, emergency physicians tend to be older, male, and White, and are less likely to have completed EM residency training or have board certification. There is also currently a higher rate of rural physicians leaving clinical practice than in urban emergency departments (ED). In this cross-sectional study we sought to identify the work environments of graduates of a rural EM residency program, and the strengths and weaknesses of such a program.

**Methods:**

We conducted a survey among 29 graduates of a community-based EM program to evaluate the effectiveness of a residency program in training physicians who will work in rural areas. The survey assessed the graduates’ perceptions of their level of preparedness, further training, and practice location after completing the program. Results are reported using descriptive statistics.

**Results:**

Twenty respondents completed the survey (69%). Most of them identified as male (60%), White (70%), and non-Hispanic or -Latino (80%). Seventy-five percent of the graduates work in counties with fewer than 1,000,000 inhabitants, and 70% work in community hospitals and EDs caring for fewer than 100,000 patients/year. Four (20%) declared to work in critical access hospitals. Overall, respondents felt confident in their residency training.

**Conclusion:**

A community-based EM training program may be an effective strategy for producing emergency physicians who go on to work in rural and smaller communities.

Population Health Research Capsule What do we already know about this issue?

*Rural communities lack board-certified emergency physicians. Residency graduates tend to practice in environments like their training environment.*

What was the research question?

*Do graduates of a community-based EM program in a rural area practice in rural areas after completing their training?*

What was the major finding of the study?

*75% of the graduates were practicing in counties with fewer than 1 million residents; 70% work in hospitals caring for fewer than 100,000 patients per year.*

How does this improve population health?

*A community-based training program in a rural area graduates emergency physicians who work in underserved, rural communities.*

## INTRODUCTION

Recent research has contributed to a greater understanding of the landscape of the emergency medicine (EM) workforce and training in the United States. In 2020, a cross-sectional study using the American Medical Association (AMA) Physician Masterfile database found that there were 48,835 clinically active emergency physicians in the US. Among them, 81% reported having EM training, and 69% were board certified in EM. The remaining 19%, who were neither EM trained nor certified, were divided among those who were family medicine- (33%), internal medicine- (24%), or surgery-trained (12%) physicians. According to the US Department of Agriculture (USDA) Urban Influence Codes (UIC) classification, 92% worked in urban areas, 6% in large rural areas, and 2% in small rural areas. Compared to urban areas, the survey found that large and small rural areas tended to be staffed by males (71% vs 80% and 81%, respectively) and older physicians (50 years of age vs 58 and 62 years, respectively), were less likely to have EM training (74% vs 52% and 37%, respectively) or board certification (70% vs 56% and 40%, respectively), and that the majority of rural emergency physicians had graduated at least 20 years before 2020.^1^

In parallel, data from the AMA Physician Masterfile and the Accreditation Council for Graduate Medical Education listing showed an increase of 105 EM residencies from 2013 to 2020. Most of these programs offer three years of training, with the median age of residents being 31 (29–33 years) and females representing 39% of the trainees. Unsurprisingly, a cross-sectional data analysis found that 98% of the 6,993 residents included in the study were receiving training in urban areas, with 77% being in places with over 1,000,000 inhabitants and 17% in areas with populations between 250,000–1,000,000 individuals. Less than 2% of the residents were training in large rural areas and 0.3% in small rural areas (urban-rural classification based on the USDA UIC).^2^

While the density of emergency physicians per 100,000 US population in urban areas has increased 1.4 times, both large and small rural areas showed a decrease of 0.4 and 3.7, respectively.^1^ One of the explanations for this phenomenon is that rural areas have a higher rate of emergency physicians leaving practice compared to urban areas.^3^ Another leading cause of this urban preference is that residents tend to practice in environments similar to where they trained and stay geographically close to their training programs, at least in the first three years after graduation.^2^ ^,^ ^4^ Given the locations of most training programs in urban centers, this does not address the rural-urban disparity in emergency care.

In this study we hypothesized that training physicians in a rural environment will make them more likely to practice in a rural area. Prior studies have demonstrated the effectiveness of alumni surveys in identifying residency problem areas to drive targeted curricular improvement.^5^ ^–^ ^7^ However, no previous studies have looked at a training program’s location in relation to its graduates’ practice patterns. As a secondary outcome, we sought to explore the specific benefits and challenges that training at a community-based program presented to its graduates.

## METHODS

The study site is in the state of New York. It is home to an EM residency training program with a 34-bed emergency department (ED) that sees an average of 35,000 visits per year and serves a largely rural population of 84,148 individuals.^8^ The first residency class began their training in 2015, and the program has graduated 29 residents in the intervening years. Due to the characteristics of the institution and residency program, its mission is to train physicians to work in rural communities with fewer than 250,000 inhabitants.

The study design was a cross-sectional anonymous survey delivered through the Survey Monkey platform (SurveyMonkey Inc, San Mateo, CA). The research protocol was reviewed by the institution’s System Review Board and deemed exempt from institutional review board approval.

All 29 graduates of the EM residency program were eligible and received the invitation to participate in the survey via emails retrieved from our contact list. The survey, consisting of 23 questions, was reviewed and amended by EM program faculty and leadership. The email text and the survey cover page instructed participants about the content and goals of the study. Agreement to answer the survey implied consent to participate in the study. The data collected included general demographics (sex, race, ethnicity, and number of years since graduation), characteristics of the graduate’s current practices (type of hospital, ED annual volume, county, if critical access), additional postgraduate training, and administrative and mentoring roles. Questions about the residency training program and how graduates feel prepared for their current practice were elaborated on a five-point Likert scale. Two open-ended questions asked about the program’s strengths and weaknesses.

The most relevant questions were placed in the first half of the survey. The survey was available for six weeks (February and March 2023), and the graduates received three email reminders to encourage them to participate.

### County Size and Urban and Rural Classifications

County sizes were reported according to the 2020 US Census Bureau.^8^ We used the USDA rural-urban continuum codes (RUCC) to categorize counties based on the population size, degree of urbanization, and proximity to a metro area (Table 1).^9^ The RUCC was suitable for this study because it has a better representation of counties smaller than 250,000 inhabitants when compared to the USDA Economic Research Service UIC used by other authors.^1^

**Table 1.** Definition of metro/non-metro US counties according to the population size, degree of urbanization, and proximity to a metro area - US Department of Agriculture Rural-Urban Continuum Codes (RUCC) (2023).^9^

Code	Classification of US counties based on the definition of metro/non-metro areas
1	Metro – counties in metro areas of 1 million population or more
2	Metro – counties in metro areas of 250,000 to 1 million population
3	Metro – counties in metro areas of fewer than 250,000 population
4	Non-metro – urban population of 20,000 or more, adjacent to a metro area
5	Non-metro – urban population of 20,000 or more, not adjacent to a metro area
6	Non-metro – urban population of 5,000 to 20,000, adjacent to a metro area
7	Non-metro – urban population of 5,000 to 20,000, not adjacent to a metro area
8	Non-metro – urban population of fewer than 5,000, adjacent to a metro area
9	Non-metro – urban population of fewer than 5,000, not adjacent to a metro area

**Figure 1.**
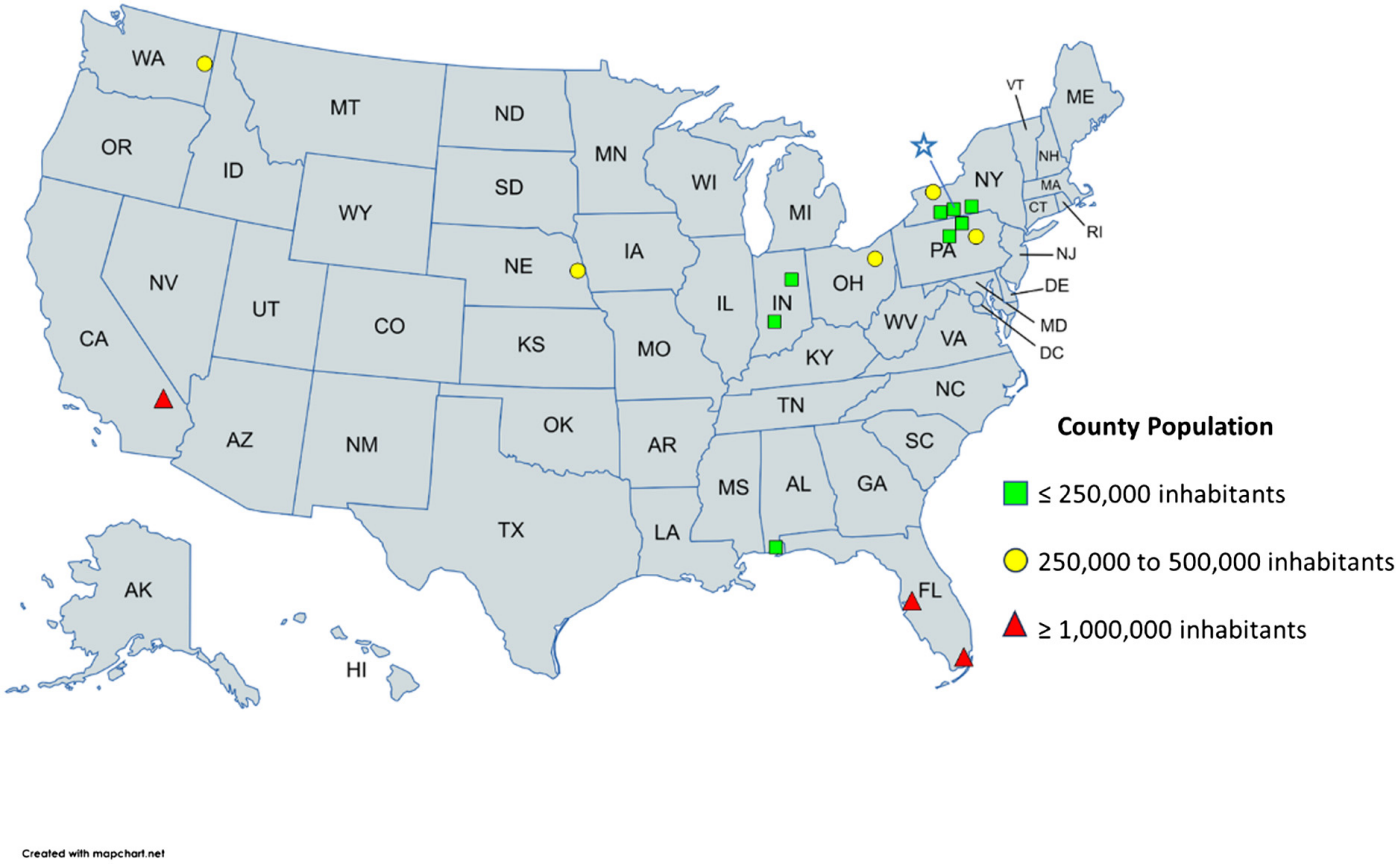
Current practices of emergency medicine graduates distributed by county population and geographic location. The blue star in the state of New York (NY) represents the study site location.

The results of this study are reported using descriptive statistics represented in tables and graphs. The United States map (Figure 1) was created using MapChart (Creative Commons Attribution 4.0 international license, Mountain View, CA) and Power Point Microsoft 365 Apps Version 2407 (Microsoft Corp, Redmond, WA).

## RESULTS

Twenty graduates completed the survey, corresponding to a 69% response rate. As presented in Table 2, most of the respondents identified themselves as male (60%), White (70%), and non-Hispanic or -Latino (80%). Nine respondents (45%) graduated after 2021 and 11 (55%) in the years before.

**Table 2.** Demographic information from the 20 residency graduates.

		Graduates	Percent
Sex	Female	7	35%
	Male	12	60%
	Other	1	5%
	Prefer not to say	0	0%
Race	White	14	70%
	Black	0	0%
	Asian	4	20%
	Native American/Alaska Native	0	0%
	Native Hawaiian/other Pacific Islander	0	0%
	Mixed Race	1	5%
	Other	1	5%
	Prefer not to say	0	0%
Ethnicity	Hispanic or Latino	1	5%
	Non-Hispanic or -Latino	16	80%
	Prefer not to say	3	15%
Years since graduation	0–2	9	45%
	> 2	11	55%

### Current Practice

Overwhelmingly, respondents self-reported working in community hospitals (14, 70%), with five working in private hospitals (25%) and one in a university hospital. Most graduates work in EDs caring for fewer than 100,000 patients/year (70%), and of those, 64% (9/14) work in EDs caring for 50,000 or fewer patients/year. Three respondents who work in community hospitals didn’t report their approximate ED annual volume. Four graduates (20%) work in critical access hospitals, according to the designation by the Centers for Medicare and Medicaid Services (Table 3).

**Table 3.** Characteristics of the counties and facilities where the resident graduates are currently practicing. The “^*^” signals respondents who declared to work in more than one type of hospital.

Study ID #	State	County of primary practice site	Type of primary practice site	Emergency department approximate annual volume	Critical access designation by the CMS	Total county population	County population living in rural areas (%)	RUCC	Distance from training site (miles)
1	NE	Douglas	University hospital/academia	60,000		584,526	2	2	721
2	NY	Monroe	Community hospital^*^	100,000–130,000	NA	759,443	8	1	83
3	NY	Chemung	Community hospital	35,000	NA	84,148	26	3	0
4	FL	Miami Dade	Private hospital	65,000	Yes	2,701,767	1	1	1,154
5	CA	San Bernardino	Private hospital	150,000	NA	2,181,654	5	1	2,238
6	NY	Broome	Community hospital	55,000	NA	198,683	25	3	42
7	IN	Monroe	Community hospital	50,000	Yes	139,718	21	3	546
8	PA	Lycoming	Community hospital	Not sure	NA	114,188	40	3	64
9	PA	Luzerne	Community hospital	Not sure	No	325,594	22	2	90
10	NY	Chemung	Private hospital	37,000	No	84,148	26	3	0
11	IN	Grant County	Community hospital	50,000	No	66,674	39	4	472
12	FL	Hillsborough	Community hospital	37,000	NA	1,459,762	4	1	1,020
13	PA	Bradford	Community hospital	Not sure	Yes	59,967	73	6	17
14	OH	Summit	Community hospital	70,000	NA	540,428	4	2	246
15	CA	San Bernardino	Private hospital	130,000		2,181,654	5	1	2,238
16	AL	Baldwin	Community hospital	28,000–35,000	NA	231,767	38	3	1,014
17	WY	--	Community hospital	4,000	Yes	--	--	--	--
18	NY	Steuben	Community hospital	34,000	No	93,584	60	4	32
19	WA	Spokane	Community hospital^*^	60,000	NA	539,339	15	2	2,001
20	PA	Lycoming	Private hospital	45,000	No	114,188	40	3	64

*CMS*, Centers for Medicare and Medicaid Services; *RUCC*, Rural-Urban Continuum Codes – Metro/non-metro classification according to the US Department of Agriculture (2023).

According to 2020 US Census Bureau data, 75% of the respondents work in counties with fewer than 1,000,000 inhabitants. Most of those (10 of 15) practice in counties with fewer than 250,000 inhabitants (codes 3, 4, and 6) and with rural populations varying from 21–73%. Forty-five percent (9/20) of the respondents live and work in the Northeast and are within 90 miles of the study institution (Table 3), and two-thirds graduated more than two years before. The blue star in Figure 1 represents the study site location in the State of New York. One physician did not report his/her county.

### Additional Training, Mentoring, and Administrative Roles

Respondents did not do another residency or change specialty areas. Three (15%) had gone on to further training, with two pursuing fellowships in addiction medicine and one pursuing fellowships in wilderness medicine and military EM, and a master’s degree in public health. Although most respondents do not work in large academic centers, all but one (95%) reported some role in mentoring or teaching medical students (75%), residents (70%), or younger colleagues (25%) since graduation. Two have or had an administrative position in the hospital where they currently work or worked previously.

### Graduates’ Impressions of Residency Training

**Figure 2.**
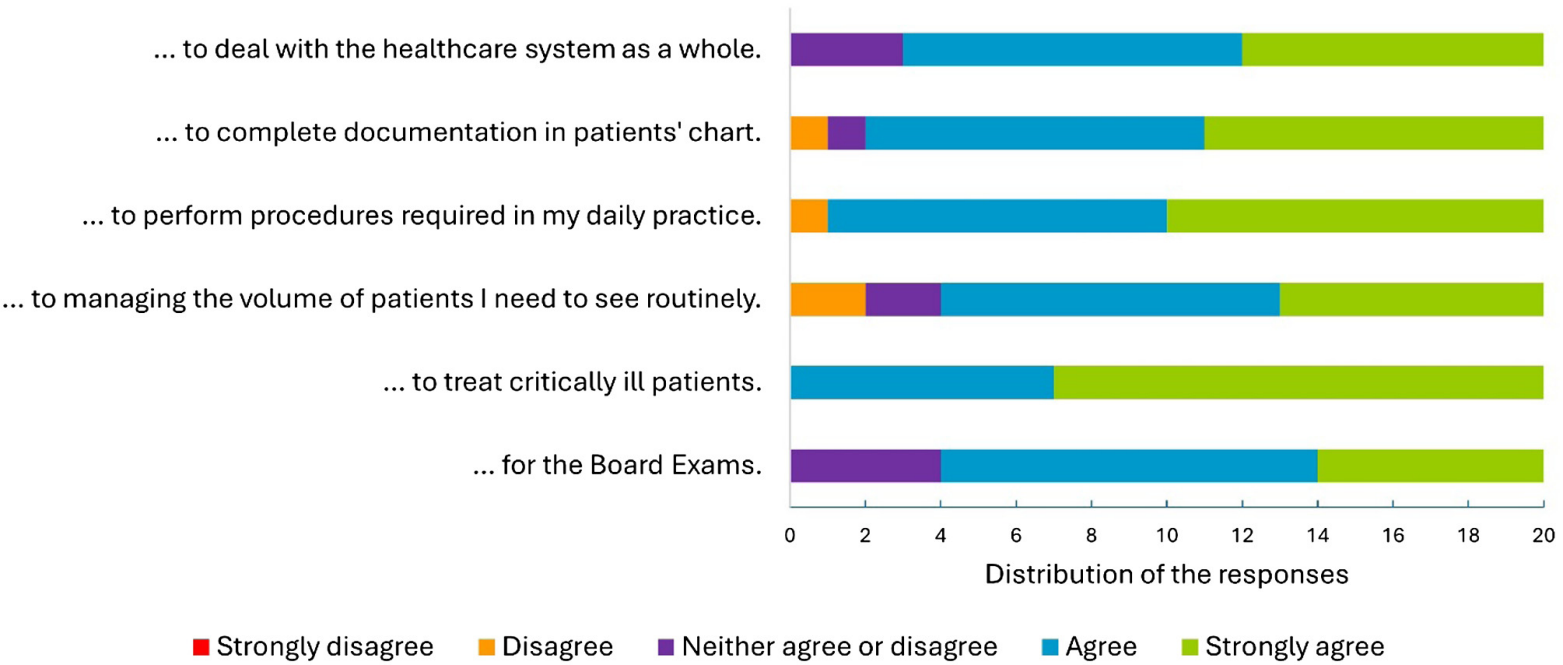
This graph represents the distribution and agreement of the 20 residents who responded to the survey with the statement, “The emergency medicine residency program prepared me well…”

Figure 2 shows the responses to the six questions in Likert-scale format about how prepared graduates felt to take the board exams and work as emergency physicians. Overall, there were positive answers to all six questions. The respondents felt very well prepared to treat critically ill patients, and no neutral or disagreeing responses were observed. With questions regarding dealing with the healthcare system and preparedness for the board exams, the responses were mainly positive but with some neutral answers. Uncommonly, graduates reported feeling somewhat less prepared for procedures necessary for daily practice (1/20) and documentation in patients’ charts (1/20). The area identified as the lowest performance of the program was related to preparing residents to manage the patient volume they need to see routinely, with two neutrals and two disagreements.

### Main Positive and Negative Aspects of the Residency Training

From the graduates’ point of view, the top three strengths and weaknesses of the EM residency program were as follows:

• Strengths: Autonomy with little competition for procedures; exposure to a good breadth of pathologies and critical care cases; one-on-one interaction with attendings.
• Weaknesses: Need for more exposure to rare procedures; more simulations/board preparation and practice with a higher volume of patients.

## DISCUSSION

To our knowledge, this is the first study to examine whether residency training in a community-based EM program in a rural area produces graduates who practice in a rural environment after graduation. The results of this study suggest that this is so, although future research should expand on these findings to look at the breadth of EM-training programs to see whether this holds true across the country. As a secondary outcome, this study highlights the effectiveness and limitations of a small, community EM residency program in training emergency physicians in general and specifically to work in underserved rural environments.

The demographics indicate that the graduates of the study site residency program were predominantly male, White, and non-Hispanic or -Latino. This distribution is similar to data from residencies around the country, regardless of whether they are large or small training programs, reflecting the need for more diversity in the EM workforce.^1^ ^,^ ^2^ ^,^ ^10^ Conscious efforts are being made to recruit residents and attendings from minorities to increase diversity and equity among emergency physicians.

Historically, emergency physicians tend to practice in environments similar to their training and to work close to their training site, at least in the first three years after graduation.^1^ ^,^ ^4^ As most residencies are located in large urban areas, graduates may feel relatively uncomfortable considering jobs in more resource-limited locations.^11^ Although the data presented here is limited, it suggests that the opposite might also be true—residents trained in small community hospitals tend to stay, and feel comfortable working in that same environment. Besides increasing the availability of rural rotations during residency or giving incentives to attract physicians to underserved areas, creating new residency programs in small urban/rural areas may be another meaningful way to reverse the trend toward a rural EM desert around the country.^1^ ^,^ ^3^ ^,^ ^11^

Other factors that attract physicians to one determined area are family and spousal ties,^4^ which can also be important for those choosing training in small programs. In this study, we did not observe a tendency of residents who graduated in the prior two years to work closer to the training program as described by other authors. This deviation could be attributed to the use of different time frames since graduation, or because of shifts in the job market since the onset of the COVID-19 pandemic.

As a secondary outcome, the survey identified areas for training improvement that could be addressed through curricular development. Overall, respondents felt confident in their residency training at this small institution. The strengths reported by the graduates were less competition for procedures, a sense of autonomy over patients, and the close resident-to-faculty relationship that had developed in a small community hospital. The main concern about training in small EDs is that residents are not exposed to sufficient volume and complex medical cases. However, a recent study comparing procedures done by second- and third-year residents training in urban vs rural centers reported a higher proportion of procedures done in large rural hospitals when data was standardized by hours worked. Residents who train with limited specialty backup learn valuable skills, such as relying on themselves to treat critically ill cases and stabilizing patients when transfers are necessary.^12^ Overall, while it was not the primary goal of this study, the results support that residents graduating from this program felt well-prepared for their EM careers.

## LIMITATIONS

The most important limitation of this study is its small sample size. The first class of graduates entered residency in 2015; therefore, the study site training program is relatively young, and the survey’s sample size is still small. However, this study serves as a pilot for future research into the practice patterns of EM residency graduates. We look forward to expanding this work in the future.

Among other study limitations are the possibilities of survey fatigue, response bias, and non-response bias. The survey had primarily multiple-choice questions, and questions related to the main goal of this study (current practice locations) were presented in the first half of the questionnaire. Thirty percent of the eligible participants did not respond to the survey. It is possible that those who did not respond felt more negatively about their training and did not want to share those reflections. Finally, it is unknown to what extent the COVID-19 pandemic affected the perceived and/or real aspects of the residency.

## CONCLUSION

The fact that 70% of the graduates of a residency program located in a rural area now work in community hospitals with an annual patient volume lower than 100,000 and 75% work in counties with fewer than 1,000,000 inhabitants suggests that this small community-based training program is succeeding in its mission. Because the nature of the program is emphasized in the recruiting material, it likely draws people with a pre-existing interest in working away from large urban areas. Access to high-quality emergency care is as critical in rural areas as it is everywhere else in the country, and this study supports that training clinicians in a rural environment fosters clinicians who are invested in the needs of rural, underserved communities.
